# Knowledge, attitudes, and practices of Chinese endocrinologists on congenital adrenal hyperplasia: emphasis on fertility management and diagnostic challenges

**DOI:** 10.3389/fpubh.2026.1733159

**Published:** 2026-03-27

**Authors:** He Sun, Li Ren, Ling Li, Ping Li

**Affiliations:** 1 Department of Endocrinology, Shengjing Hospital of China Medical University, Shenyang, Liaoning, China; 2 Department of Cardiology, Shengjing Hospital of China Medical University, Shenyang, China

**Keywords:** congenital adrenal hyperplasia, endocrinologists, knowledge, attitude, practice, polycystic ovary syndrome, reproductive health

## Abstract

**Introduction:**

This study aimed to evaluate the knowledge, attitudes, and practices (KAP) of Chinese endocrinologists regarding congenital adrenal hyperplasia (CAH), which is essential for enhancing clinical differentiation skills and improving fertility management in non-classic CAH patients.

**Methods:**

This cross-sectional study was conducted between July 27 and August 23, 2023, using a questionnaire distributed to 475 endocrinologists across China. KAP levels were assessed using a 70% maximum score threshold for descriptive purposes, and factors associated with KAP were identified via multivariate logistic regression.

**Results:**

The median scores were 21/27 (knowledge), 35/40 (attitudes), and 28/35 (practices). Despite positive attitudes, significant gaps in fertility-related knowledge were evident: nearly 30% of respondents failed to recognize that female CAH patients could achieve natural pregnancy through proper individualized treatment, and 24% failed to identify fertility preservation as a key treatment goal. Diagnostic confidence was also limited (53.9%), with over half of participants unclear on the most common enzyme deficiencies crucial for differential diagnosis. Logistic regression analysis revealed that having a doctoral degree, an intermediate or senior professional rank, and experience in diagnosing CAH were associated with more proactive clinical practices, especially in patient education and reproductive health counseling.

**Conclusion:**

While Chinese endocrinologists demonstrate generally positive attitudes toward CAH, significant knowledge and practice gaps persist, particularly in fertility management and diagnostic differentiation. The limited awareness of reproductive outcomes and low diagnostic confidence highlight an urgent need for targeted education to improve individualized fertility treatment options and reduce misdiagnosis in clinical practice.

## 1 Introduction

Congenital adrenal hyperplasia (CAH) is a chronic, inherited disorder that affects adrenal steroid biosynthesis, primarily due to congenital enzyme deficiencies in key adrenal corticosteroid biosynthetic pathways, such as 21-hydroxylase (90%–95%), 11β-hydroxylase (5%–8%), and 17α-hydroxylase (approximately 1%) (1–3). Clinically, 21-OHD is categorized into classic and non-classic (NCCAH) forms. The classic form is further divided into the salt-wasting (SW) type, presenting with life-threatening cortisol and aldosterone deficiency alongside androgen excess, and the simple virilizing (SV) type, which lacks severe salt-wasting but causes significant prenatal virilization in females. In contrast (4), NCCAH is a milder, late-onset variant where patients produce sufficient aldosterone and cortisol but manifest symptoms of postnatal androgen excess (5). While 21-OHD is characterized by hyperandrogenism and potential mineralocorticoid deficiency, 11β-OHD leads to both hyperandrogenism and mineralocorticoid excess with hypertension; conversely, 17α-hydroxylase deficiency uniquely results in sex hormone deficiency alongside mineralocorticoid-induced hypertension (6). CAH affects approximately 1 in 10,000 to 20,000 live births globally, with varying prevalence by region and genotype (7, 8).

Early and accurate diagnosis is pivotal for effective CAH management, particularly for preserving fertility. Treatment typically necessitates lifelong glucocorticoid replacement, which, when optimized, can suppress excess androgens in 21-OHD and 11β-OHD and prevent early-onset puberty. However, adult patients across all subtypes face significant reproductive hurdles. In female patients, NCCAH frequently presents with hirsutism and ovarian dysfunction, clinical features that overlap significantly with Polycystic Ovary Syndrome (PCOS), leading to frequent misdiagnosis and missed windows for fertility intervention. Conversely, 17α-hydroxylase deficiency results in sex hormone deficiency, leading to primary amenorrhea in females and male pseudohermaphroditism. In male patients with hyperandrogenic subtypes, particularly those with the classic forms (SW and SV), the persistent elevation of ACTH can drive the development of testicular adrenal rest tumors (TARTs) (9, 10). These tumors are a major cause of infertility as they can obstruct seminiferous tubules and impair spermatogenesis (11). These gender-specific reproductive complications highlight the importance of enhancing clinical awareness and diagnostic accuracy for various CAH subtypes in both female and male patients, with particular emphasis on distinguishing CAH from PCOS in women and monitoring for TART in men (9, 10).

The limited awareness of CAH among healthcare providers often leads to diagnostic difficulties and delays, increasing the risk of long-term complications for patients. In clinical practice, endocrinologists play a crucial role in the early identification and long-term management of CAH. Although existing Knowledge-Attitude-Practice (KAP) surveys have primarily centered around patient awareness and adherence (12), there is a noticeable lack of attention to the diagnostic and management capabilities of healthcare providers. In China, the current status of CAH diagnosis faces numerous challenges: female patients are often misdiagnosed with PCOS, while male patients lack standardized screening for TARTs, leading to diagnostic delays and impaired fertility outcomes. These sex-specific diagnostic challenges not only hinder timely intervention but also lead to suboptimal reproductive outcomes. Despite international advancements in individualized treatment strategies for reproductive health in CAH patients, such progress has not been uniformly reflected in Chinese clinical practice (13). These discrepancies underscore an urgent need for systematic assessment of physicians’ knowledge and clinical behaviors. Evaluating endocrinologists’ KAP is essential not only to identify gaps in clinical competency but also to inform targeted educational initiatives and policy reforms, with the ultimate goal of improving diagnostic accuracy and enhancing fertility outcomes in CAH patients. This study thus aims to bridge this critical gap, with particular emphasis on fertility preservation and diagnostic differentiation, to ultimately enhance reproductive outcomes and quality of care for CAH patients in China.

## 2 Materials and methods

### 2.1 Study design and participants

This cross-sectional study was conducted from July 27, 2023, to August 23, 2023. A total of 475 endocrinologists from hospitals across all seven major geographical regions (Northeast, East, North, Central, South, Southwest, and Northwest China) participated in the survey. Among the respondents, most were affiliated with public tertiary hospitals (388/475, 81.7%), while the remainder were from other hospital tiers (87/475, 18.3%). The study’s inclusion criteria encompassed adult endocrinologists who willingly participated in the research and provided informed consent. Exclusion criteria included respondents who declined to participate, those who completed questionnaires in less than 120 s or more than 1,800 s, respondents with incomplete or duplicate questionnaires, and questionnaires that contained logical errors, such as abnormally entered age. This study was approved by the Ethics Committee of Shengjing Hospital of China Medical University (2023PS986K), and all participants provided written informed consent.

### 2.2 Data collection

The questionnaire was administered via the WeChat-based “Questionnaire Star” platform and distributed through verified professional WeChat academic groups composed of endocrinologists nationwide. These groups included national and provincial endocrinology associations, academic committee groups, subspecialty working groups, and training physician groups. Many of the groups were initiated and managed by senior academic leaders, and membership required professional verification to ensure that participants were qualified endocrinology professionals. To ensure broad geographical and institutional representation and to minimize selection and regional bias, we leveraged a multi-layered professional network stratified by geographical regions (Northeast, East, North, Central, South, Southwest, and Northwest China), healthcare institution levels (provincial tertiary hospitals, municipal general hospitals, and county-level hospitals), and professional affiliations. The survey link was initially shared by department directors or senior clinicians and further disseminated within their professional networks. Participation was entirely voluntary, and no incentives were provided. To prevent duplicate entries, the platform was configured to allow only one submission per WeChat account and device. This multi-layered distribution strategy ensured participation from endocrinologists across different administrative levels, institutional grades, and regions of China. The recruitment also included residents in endocrinology training programs and practicing endocrinologists in primary and secondary care facilities. Through this comprehensive approach, the study maximized coverage and minimized the risk of over-representation from specific hospital tiers or geographic locations.

### 2.3 Questionnaires

The questionnaire was developed with reference to previously published literature (7, 14–16) and “Diagnosis and Management of Congenital Adrenal Hyperplasia” (17), and underwent revisions based on feedback provided by a panel of experts, comprising three distinguished endocrinologists, a reproduction specialist, and an obstetrics and gynecology expert. The reproductive health specialists primarily offered insights on male and female CAH-related fertility concerns, while the obstetrics and gynecology expert focused on advice regarding the differential diagnosis of PCOS. The three endocrinology experts provided valuable recommendations on various aspects, including CAH symptoms, diagnosis, differentiation, and treatment. Rigorous efforts were made to enhance the questionnaire’s clarity and comprehensibility through three expert discussions, resulting in the removal of redundant or similar items.

The questionnaire, written in Chinese, consisted of four distinct sections (appendix): demographic characteristics (9 items), knowledge (15 items), attitudes (8 items), and practices (7 items). Separate scores were calculated for each subcategory, with each section having a predefined maximum score: 15 for knowledge, 40 for attitudes, and 35 for practices. For the knowledge section, responses were coded as either 1 for “true” or 0 for “false” or “not sure.” The attitudes section was evaluated using a five-point Likert scale, ranging from “totally agree” (5) to “totally disagree” (1). Negative-topic scores were reverse-coded prior to calculation. Similarly, the practice section was assessed using a five-point Likert scale, with responses ranging from “always” (5) to “never” (1). Adequate knowledge, positive attitudes, and proactive practices were defined as achieving scores surpassing 70% of the maximum possible score in each respective section (18). Subsequently, a pilot study involving 39 randomly selected endocrinologists was conducted to assess the questionnaire’s reliability, yielding a robust Cronbach’s *α* coefficient value of 0.949. To further assess the construct validity of the questionnaire, an exploratory factor analysis (EFA) was conducted on the full sample. The Kaiser-Meyer-Olkin (KMO) measure of sampling adequacy was 0.894, indicating suitability for factor analysis. Bartlett’s test of sphericity was statistically significant (*p* < 0.001), supporting the factorability of the correlation matrix. These results confirmed that the scale structure was appropriate for evaluating the KAP dimensions.

### 2.4 Statistical analysis

The sample size required for the questionnaire survey was calculated using the Open Epi-calculator with the specific formula $=\frac{p\times (1-p)\times z^{2}}{e^{2}}$ , based on this formula to obtain the needed sample size for the survey (19, 20). Among them:


$$n=\frac{0.5\times (1-0.5)\times 1.96^{2}}{0.05^{2}}=384.16$$

The result calculated by the formula is 384, so collecting at least 384 valid questionnaires is the minimum sample size required to ensure the credibility of the research results.

Data analysis was conducted using SPSS 22.0 (IBM, Armonk, NY, USA). Continuous data are presented as the median (25%–75% percentiles), while categorical data are expressed as n (%). Continuous variables underwent a normality test, with the t-test for normally distributed data and the Wilcoxon Mann–Whitney test for non-normally distributed data when comparing two groups. For three or more groups with normally distributed continuous variables and uniform variance, ANOVA was used for comparisons, while the Kruskal–Wallis test was employed for non-normally distributed data. For multivariate analysis, binary logistic regression was performed to identify factors independently associated with adequate knowledge, positive attitudes, and proactive practices. Each KAP domain was dichotomized using its respective median score as the threshold. Variables with a univariate *p*-value <0.1 were included in the multivariate model. Odds ratios (ORs) and 95% confidence intervals (CIs) were reported for each variable. A two-sided *p*-value less than 0.05 was considered statistically significant.

## 3 Results

### 3.1 Demographics characteristics

A total of 505 questionnaires were initially collected. After applying the predefined exclusion criteria—including completion time of less than 120 s or more than 1,800 s, incomplete questionnaires, logically inconsistent responses (such as abnormal age entries), and incorrect hospital information—475 valid questionnaires were included in the final analysis. The 475 valid questionnaires (Cronbach’s *α* = 0.878, KMO = 0.894) from predominantly female endocrinologists (82.3%) in public tertiary hospitals (81.7%), with 61.3% holding senior professional ranks and 51.2% having >15 years of clinical experience (Table 1).

**Table 1:** Demographic information.

	*N* (%)	Knowledge (K)		Attitude (A)		Practice (P)	
		Median (25%–75% percentiles)	*p*	Median (25%–75% percentiles)	*p*	Median (25%–75% percentiles)	*p*
Total	475	21 (19, 24)		35 (32, 36)		28 (25, 30)	
Gender			0.251		0.260		0.025
Male	84 (17.7)	21 (18, 24)		34 (32, 36)		27.5 (24, 29)	
Female	391 (82.3)	22 (19, 24)		35 (33, 36)		28 (26, 30)	
Age			<0.001		0.037		0.298
≤30 years	69 (14.5)	19 (14, 21)		34 (30, 35)		27 (24, 30)	
31–40 years	139 (29.3)	22 (20, 24)		35 (33, 36)		28 (24, 30)	
41–50 years	158 (33.3)	22 (20, 25)		35 (33, 36)		28 (26, 30)	
>50 years	109 (22.9)	22 (19, 24)		35 (33, 36)		28 (25, 30)	
Education level			<0.001		<0.001		<0.001
Bachelor’s degree	159 (33.5)	20 (17, 22)		34 (31, 36)		27 (24, 29)	
Master’s degree	179 (37.7)	22 (19, 24)		34 (32, 36)		28 (25, 30)	
Doctoral degree	137 (28.8)	23 (21, 26)		35 (34, 36)		29 (27, 30)	
Professional role			<0.001		0.066		0.296
Endocrinologists	434 (91.4)	22 (19, 24)		35 (33, 36)		28 (25, 30)	
Endocrinology resident	41 (8.6)	19 (14, 21)		34 (30, 36)		27 (24, 30)	
Professional rank			<0.001		0.014		0.031
No professional rank	35 (7.4)	19 (13, 21)		33 (29, 35)		26 (22, 29)	
Junior-level rank	36 (7.6)	20 (17.5, 23)		35 (32.5, 36)		27 (24, 30)	
Intermediate-level rank	113 (23.8)	21 (19, 23)		35 (32, 36)		29 (25, 30)	
Senior-level rank	291 (61.3)	22 (20, 25)		35 (33, 36)		28 (26, 30)	
Years of work experience			<0.001		0.195		0.108
≤3 years	64 (13.5)	19 (14.5, 22)		34 (30, 35.5)		26.5 (24, 30)	
3–5 years	17 (3.6)	20 (20, 21)		35 (33, 36)		29 (24, 30)	
5–10 years	69 (14.5)	22 (19, 24)		35 (32, 36)		29 (26, 30)	
10–15 years	82 (17.3)	22 (20, 24)		34.5 (33, 36)		28 (25, 30)	
>15 years	243 (51.2)	22 (20, 24)		35 (33, 36)		28 (26, 30)	
Years of work experience variable adjustment			<0.001		0.163		0.099
<5 years	81 (17.1)	20 (16, 22)		35 (30, 36)		27 (24, 30)	
5–10 years	69 (14.5)	22 (19, 24)		35 (32, 36)		29 (26, 30)	
10–15 years	82 (17.3)	22 (20, 24)		34.5 (33, 36)		28 (25, 30)	
>15 years	243 (51.2)	22 (20, 24)		35 (33, 36)		28 (26, 30)	
Hospital type			<0.001		0.007		0.174
Public primary	10 (2.1)	19 (16, 26)		32.5 (31, 35)		27 (26, 30)	
Public secondary	47 (9.9)	19 (17, 22)		34 (31, 36)		27 (24, 30)	
Public tertiary	388 (81.7)	22 (19.5, 24)		35 (33, 36)		28 (26, 30)	
Private hospitals	30 (6.3)	21 (17, 22)		33.5 (30, 35)		27 (25, 29)	
Hospital type variable adjustment			<0.001		0.001		0.032
Public tertiary	388 (81.7)	22 (19.5, 24)		35 (33, 36)		28 (26, 30)	
Other hospital	87 (18.3)	20 (16, 22)		34 (31, 36)		27 (24, 30)	
Number of CAH patients you have diagnosed or treated			<0.001		<0.001		0.058
0	207 (43.6)	20 (17, 23)		34 (32, 36)		28 (24, 30)	
1–5	164 (34.5)	22 (20, 24)		35 (33, 36)		28 (26, 30)	
6–10	46 (9.7)	24 (21, 26)		35 (33, 36)		29 (27, 30)	
11–20	26 (5.5)	25 (22, 26)		36 (34, 37)		28 (26, 29)	
>20	32 (6.7)	22 (20, 25.5)		35 (33.5, 36)		27.5 (26, 29)	

### 3.2 Knowledge gaps and disparities

Participants demonstrated selective competency in CAH knowledge, with a median score of 21/27 (25th–75th percentile: 19–24). Critical gaps directly impacting fertility management and diagnosis were prominent. For instance, 24% failed to acknowledge that preserving fertility is a key treatment goal (K15), and over half (55.6%) could not correctly identify 21-hydroxylase deficiency as the predominant subtype (K3), a crucial step in differential diagnosis from PCOS, reflecting poor recognition of 21-hydroxylase deficiency as the predominant subtype. Furthermore, while 89.9% recognized female masculinization (K6), merely 45.9% understood that CAH could induce male feminization (K7), indicating a limited understanding of different enzymatic subtypes. Fundamental misconceptions also persisted, with 48.4% erroneously attributing CAH to autosomal dominant inheritance (K1) (Supplementary Table S1).

### 3.3 Attitudinal paradoxes

Attitudinal assessment, with a median score of 35/40 (25%–75% percentile: 32–36). A key contradiction emerged between diagnostic vigilance and self-perceived capability. While 94.5% recognized CAH’s susceptibility to misdiagnosis (A1: 64.8% strongly agree + 29.7% agree), only 53.9% expressed confidence in clinically identifying it (P7). Regarding fertility, while a notable majority (70.7%) agreed that female patients could achieve natural pregnancy with individualized treatment (A4: 30.3% strongly agree + 40.4% agree), nearly 30% of endocrinologists still harbor doubts or are uncertain about this key reproductive outcome. Moreover, concerning therapeutic practices during pregnancy, 52.4% inappropriately approved the continuous use of dexamethasone (A5: 19.6% strongly agree + 32.8% agree), directly conflicting with international guidelines (Supplementary Table S2).

### 3.4 Practice patterns and disconnects

Practice patterns, with a median score of 28/35 (25%–75% percentile: 25–30), exposed systemic disconnects. Diagnostic hesitancy permeated clinical work, with 41.5% uncertain about identifying CAH (P7) despite 75.8% advocating multidisciplinary collaboration (P6). Surgical conservatism emerged as only 44.4% consistently recommended testicular removal for CAH patients whose social gender is female (P3). Counterintuitively, intermediate practitioners demonstrated stronger protocol adherence than senior counterparts (OR = 3.032 vs. 2.844, *p* = 0.008), independent of case exposure (Supplementary Table S3).

### 3.5 Factors associated with KAP scores

Correlation analysis indicated that significant positive correlations were found between knowledge and attitudes (*r* = 0.378, *p* < 0.001), as well as practice (*r* = 0.293, *p* < 0.001). Moreover, there was also correlation between attitudes and practice (*r* = 0.471, *p* < 0.001) (Table 2).

**Table 2:** Correlation analysis.

	Knowledge	Attitude	Practice
Knowledge	1.000	0.378 (*p* < 0.001)	0.293 (*p* < 0.001)
Attitude	0.378 (*p* < 0.001)	1.000	0.471 (*p* < 0.001)
Practice	0.293 (*p* < 0.001)	0.471 (*p* < 0.001)	1.000

Multivariate logistic regression showed that age, education level, and number of CAH patients they have diagnosed or treated were independently associated with good knowledge. Specifically, those endocrinologists who are 31–40 years old (vs. < 30 OR = 4.567, 95% CI: [2.324–8.977], *p* < 0.001), 41–50 years old (vs. < 30 OR = 5.548, 95% CI: [2.766–11.130], *p* < 0.001), 50 years and above (vs. < 30 OR = 3.618, 95% CI: [1.698–7.708], *p* < 0.001), having a doctoral degree (OR = 2.512, 95% CI: [1.369–4.607], *p* = 0.003), and have diagnosed or treated 11–20 CAH patients (OR = 3.932, 95% CI: [1.202–12.860], *p* = 0.024) had higher knowledge scores (Table 3). Meanwhile, having a doctoral degree was also independently associated with positive attitude (OR = 1.940, 95% CI: [1.169–3.217], *p* = 0.010) (Table 4) and proactive practice (OR = 2.094, 95% CI: [1.302–3.367], *p* = 0.002). Furthermore, practice was also independently affected by professional rank, but it is worth noting that those with intermediate title (vs. None OR = 3.032, 95% CI: [1.342–6.850], *p* = 0.008) had a higher probability of proactive practice than those with senior title (vs. None OR = 2.844, 95% CI: [1.321–6.124], *p* = 0.008) (Table 5).

**Table 3:** Univariate and multivariate regression analysis of knowledge dimension.

Cut-off value: ≥21/<21		Univariate		Multivariate (forward method, *p* < 0.1)	
	No.	OR (95% CI)	*p*	OR (95% CI)	*p*
Gender					
Male	46/84	ref.			
Female	240/391	1.313 (0.816, 2.112)	0.262		
Age					
≤30 years	19/69	ref.		ref.	
31–40 years	89/139	4.684 (2.491, 8.810)	<0.001	4.567 (2.324, 8.977)	<0.001
41–50 years	112/158	6.407 (3.413, 12.030)	<0.001	5.548 (2.766, 11.130)	<0.001
>50 years	66/109	4.039 (2.102, 7.761)	<0.001	3.618 (1.698, 7.708)	0.001
Education level					
Bachelor’s degree	71/159	ref.		ref.	
Master’s degree	109/179	1.930 (1.251, 2.977)	0.003	2.442 (1.482, 4.025)	<0.001
Doctoral degree	106/137	4.238 (2.550, 7.043)	<0.001	2.512 (1.369, 4.607)	0.003
Professional role					
Endocrinologists	273/434	ref.			
Endocrinology resident	13/41	0.274 (0.138, 0.544)	<0.001		
Professional rank					
No professional rank	10/35	ref.			
Junior-level rank	14/36	1.591 (0.589, 4.296)	0.360		
Intermediate-level rank	62/113	3.039 (1.336, 6.912)	0.008		
Senior-level rank	200/291	5.495 (2.534, 11.916)	<0.001		
Years of work experience					
<5 years	29/81	ref.			
5–10 years	45/69	3.362 (1.717, 6.584)	<0.001		
10–15 years	55/82	3.653 (1.913, 6.975)	<0.001		
>15 years	157/243	3.273 (1.937, 5.533)	<0.001		
Hospital type					
Public tertiary	248/388	ref.			
Other hospital	38/87	0.438 (0.273, 0.702)	0.001		
Number of CAH patients you have diagnosed or treated					
0	89/207	ref.		ref.	
1–5	115/164	3.112 (2.018, 4.798)	<0.001	2.107 (1.296, 3.425)	0.003
6–10	37/46	5.451 (2.502, 11.875)	<0.001	2.906 (1.207, 6.994)	0.017
11–20	22/26	7.292 (2.427, 21.914)	<0.001	3.932 (1.202, 12.860)	0.024
>20	23/32	3.388 (1.495, 7.680)	0.003	1.895 (0.745, 4.820)	0.179

**Table 4:** Univariate and multivariate regression analysis of attitude dimension.

Cut-off value: ≥35/<35		Univariate		Multivariate (forward method, *p* < 0.1)	
	No.	OR (95% CI)	*p*	OR (95% CI)	*p*
Gender					
Male	37/84	ref.			
Female	206/391	1.414 (0.880, 2.273)	0.152		
Age					
≤ 30 years	32/69	ref.			
31–40 years	73/139	1.279 (0.717, 2.281)	0.405		
41–50 years	83/158	1.280 (0.726, 2.256)	0.394		
> 50 years	55/109	1.178 (0.644, 2.155)	0.596		
Education level					
Bachelor’s degree	69/159	ref.		ref.	
Master’s degree	86/179	1.206 (0.785, 1.853)	0.392	1.037 (0.657, 1.637)	0.875
Doctoral degree	88/137	2.343 (1.465, 3.746)	<0.001	1.940 (1.169, 3.217)	0.010
Professional role					
Endocrinologists	224/434	ref.			
Endocrinology resident	19/41	0.810 (0.426, 1.539)	0.519		
Professional rank					
No professional rank	14/35	ref.			
Junior-level rank	20/36	1.875 (0.730, 4.815)	0.191		
Intermediate-level rank	57/113	1.527 (0.707, 3.298)	0.282		
Senior-level rank	152/291	1.640 (0.803, 3.351)	0.175		
Years of work experience					
<5 years	41/81	ref.			
5–10 years	37/69	1.128 (0.593, 2.146)	0.713		
10–15 years	41/82	0.976 (0.528, 1.803)	0.937		
>15 years	124/243	1.017 (0.615, 1.681)	0.949		
Hospital type					
Public tertiary	211/388	ref.		ref.	
Other hospital	32/87	0.488 (0.302, 0.788)	0.003	0.594 (0.352, 1.003)	0.051
Number of CAH patients you have diagnosed or treated					
0	90/207	ref.			
1–5	89/164	1.543 (1.022, 2.330)	0.039		
6–10	29/46	2.218 (1.148, 4.285)	0.018		
11–20	18/26	2.925 (1.217, 7.031)	0.016		
>20	17/32	1.473 (0.698, 3.109)	0.309		

**Table 5:** Univariate and multivariate regression analysis of practice dimension.

Cut-off value: ≥28 /<28		Univariate		Multivariate (forward method, *p*<0.1)	
	No.	OR (95% CI)	*p*	OR (95% CI)	*p*
Gender					
Male	42/84	ref.			
Female	227/391	1.384 (0.863, 2.220)	0.178		
Age					
≤30 years	32/69	ref.			
31–40 years	80/139	1.568 (0.877, 2.802)	0.129		
41–50 years	96/158	1.790 (1.012, 3.168)	0.046		
> 50 years	61/109	1.469 (0.802, 2.693)	0.213		
Education level					
Bachelor’s degree	76/159	ref.		ref.	
Master’s degree	102/179	1.447 (0.942, 2.223)	0.092	1.679(1.074, 2.625)	0.023
Doctoral degree	91/137	2.160 (1.348, 3.463)	0.001	2.094(1.302, 3.367)	0.002
Professional role					
Endocrinologists	251/434	ref.			
Endocrinology resident	18/41	0.571 (0.299, 1.088)	0.088		
Professional rank					
No professional rank	12/35	ref.		ref.	
Junior-level rank	17/36	1.715 (0.659, 4.464)	0.269	1.705(0.648, 4.487)	0.280
Intermediate-level rank	68/113	2.896 (1.310, 6.401)	0.009	3.032(1.342, 6.850)	0.008
Senior-level rank	172/291	2.770 (1.327, 5.783)	0.007	2.844(1.321, 6.124)	0.008
Years of work experience					
<5 years	35/81	ref.			
5–10 years	44/69	2.313 (1.197, 4.471)	0.013		
10–15 years	50/82	2.054 (1.100, 3.835)	0.024		
>15 years	140/243	1.786 (1.075, 2.969)	0.025		
Hospital type					
Public tertiary	228/388	ref.			
Other hospital	41/87	0.625 (0.392, 0.998)	0.049		
Number of CAH patients you have diagnosed or treated					
0	106/207	ref.			
1–5	96/164	1.345 (0.890, 2.034)	0.160		
6–10	32/46	2.178 (1.098, 4.319)	0.026		
11–20	19/26	2.586 (1.043, 6.415)	0.040		
>20	16/32	0.953 (0.453, 2.006)	0.899		

## 4 Discussion

This study provides a comprehensive assessment of the current knowledge, attitudes, and practices of Chinese endocrinologists concerning CAH, a complex disorder requiring nuanced clinical management. Our findings reveal a multifaceted landscape: while endocrinologists demonstrated generally positive attitudes towards managing CAH and a willingness to engage in proactive practices, significant and specific gaps were identified in their knowledge base, particularly regarding fertility preservation strategies and the nuances of enzymatic deficiencies. Furthermore, notable discrepancies emerged between expressed attitudes and reported diagnostic confidence, as well as between the perceived importance of certain practices and their consistent implementation. These observations highlight the necessity for targeted educational interventions to bridge the gap between awareness and proficient clinical application.

The study illuminates the varying levels of knowledge among Chinese endocrinologists concerning CAH, with certain misconceptions and knowledge gaps persisting, particularly in aspects related to fertility preservation and reproductive outcomes. Regarding errors in sex assignment, nearly 90% of participants could identify that CAH can cause masculinization of females, while less than half were aware that CAH can also cause feminization of males. This significant disparity suggests that endocrinologists possess a skewed understanding of CAH enzymatic subtypes, primarily focusing on those characterized by hyperandrogenism. Specifically, while clinicians are familiar with 21-hydroxylase deficiency (21-OHD) and 11β-hydroxylase deficiency (11β-OHD)—both of which lead to excess androgen production, resulting in female virilization (pseudohermaphroditism) and male precocious puberty—there is a profound lack of awareness regarding 17α-hydroxylase deficiency. In this rarer subtype, the biosynthesis of sex hormones is impaired, leading to diametrically opposite clinical manifestations: 46, XY males often present with male pseudohermaphroditism, characterized by female-appearing external genitalia and a lack of masculinization (often resulting in them being raised as females), while 46, XX females exhibit primary amenorrhea and a failure to develop secondary sexual characteristics (such as breast development and pubic hair) during puberty (21). The inability to distinguish between these hyperandrogenic and hypoandrogenic forms of CAH could lead to severe delays in diagnosis and inappropriate social gender assignment or management. Additionally, endocrinologists exhibited significant misconceptions regarding the genetic nature of CAH and its association with adrenal malignancy. While CAH is an autosomal recessive disorder caused by mutations in genes such as CYP21A2, nearly half of the respondents in this study incorrectly believed it follows an autosomal dominant inheritance pattern. Furthermore, the finding that 48.4% of participants erroneously attributed CAH to an autosomal dominant inheritance pattern is particularly concerning. This fundamental misconception could have direct consequences for genetic counseling and family planning guidance. As an autosomal recessive (AR) disorder, CAH carries a 25% risk of being transmitted to offspring in each pregnancy when both parents are carriers. Misidentifying the inheritance pattern as dominant may lead to profound inaccuracies in offspring risk assessment—either by overestimating the risk to the patient’s children or by failing to emphasize the necessity of carrier screening for partners. Such gaps in knowledge hinder the ability of endocrinologists to provide precise reproductive counseling, which is essential for informed decision-making regarding prenatal diagnosis and pre-implantation genetic testing. Similarly, some endocrinologists mistakenly associated CAH with an elevated risk of adrenal malignancy, despite existing evidence showing that most adrenal masses in CAH patients such as adrenal myelolipomas are benign (14). Studies from Europe and North America also report knowledge gaps among physicians, though with regional variation. For instance, a survey among U.S. adult endocrinologists revealed limited confidence in providing genetic counseling for CAH patients and low familiarity with long-term tumor risk profiles (12). These findings collectively highlight the need for improved training in genetic principles and adrenal imaging interpretation in CAH care. These gaps underscore the need for targeted educational programs, continuous medical education, and interdisciplinary collaboration to ensure accurate and up-to-date knowledge (22, 23). In an ever-evolving field like healthcare, initiatives to bridge these knowledge deficiencies are crucial for delivering quality care to CAH patients. Moreover, enhancing awareness of the goals of modern adult CAH management, aimed at preserving normal reproductive function, is essential for effective patient care (24). By addressing these knowledge shortcomings, we can significantly improve clinical practice and patient outcomes in the management of CAH. A critical finding of this study is the diagnostic uncertainty among endocrinologists, which may stem from the distinct clinical profiles of classic and non-classic CAH (NCCAH). Classic CAH, often diagnosed in infancy, requires intensive management of salt-wasting and adrenal crises, whereas NCCAH frequently presents in adolescence or adulthood with symptoms mimicking polycystic ovary syndrome (PCOS), such as hirsutism and irregular menses (25). Our results showed that over half of the participants were unclear on 21-hydroxylase deficiency being the predominant enzyme deficiency, which is particularly problematic for identifying NCCAH in female patients (26). Furthermore, while fertility preservation was recognized as a goal, the management strategies differ significantly: classic CAH requires meticulous glucocorticoid titration to balance androgen suppression and growth, whereas NCCAH management focuses on restoring ovulation or improving spermatogenesis (3). The lack of confidence in identifying CAH observed in our cohort likely reflects the challenge of distinguishing NCCAH from more prevalent disorders like PCOS. Our findings regarding male CAH complications require nuanced interpretation across subtypes. TART is a hallmark complication primarily observed in classic CAH (especially the SW type) due to chronic ACTH overstimulation of adrenal-like tissue within the testes. In contrast, TART is significantly less common in NCCAH. While nearly 75% of our participants advocated for multidisciplinary collaboration, the low confidence in diagnosing CAH may stem from these distinct clinical trajectories: the challenge of managing life-long replacement and TART in classic patients versus the challenge of distinguishing NCCAH from PCOS in female adults. The finding that nearly 30% of endocrinologists failed to recognize the possibility of natural pregnancy in female CAH patients with individualized treatment highlights a critical deficiency in reproductive health knowledge. This disparity in diagnostic proficiency may be attributed to a ‘frequency bias,’ where the high clinical prevalence of PCOS leads practitioners to overlook rarer underlying etiologies like NCCAH during routine evaluations. Furthermore, while classic CAH is initially managed by pediatricians, patients are typically transitioned to adult endocrinology after age 14 for long-term surveillance. The observed knowledge gap in managing classic CAH does not necessarily stem from a lack of clinical exposure, but rather from a deficiency in proactive screening protocols and nuanced hormonal management during the transition and adult years. Long-term reproductive complications, such as TART in males, often remain asymptomatic during childhood and may only manifest as irreversible infertility in adulthood if not strictly monitored. Our findings suggest that adult endocrinologists may under-recognize the critical window for intervention, highlighting a need for standardized protocols to ensure continuity of care and preservation of reproductive function. This misperception can directly impact patient counseling and delay appropriate fertility interventions. International studies have also reported similar knowledge gaps. For example, a U.S.-based survey (12) found that many adult endocrinologists lacked confidence in managing fertility issues among CAH patients during the transition to adult care. In European settings, although fertility outcomes have improved with optimized glucocorticoid therapy and assisted reproductive technologies, clinician awareness remains inconsistent (2, 3). These findings underscore the global need for continuing education programs that emphasize individualized fertility management and reproductive potential in CAH, particularly in non-classic forms that are frequently misdiagnosed as PCOS.

The results of this study shed light on the attitudes of Chinese endocrinologists towards CAH, revealing a generally positive disposition, particularly regarding the importance of fertility preservation and reproductive health management. Notably, a substantial proportion strongly agree or agree with the importance of various aspects related to CAH diagnosis and treatment, such as the risk of misdiagnosis and underdiagnosis, the significance of 17-*α*-hydroxyprogesterone in differentiation from other conditions, the lifelong oral corticosteroid treatment for confirmed CAH, and the potential for natural pregnancy after individualized treatment. However, there are specific areas where attitudes require targeted improvement. Notably, over half of the surveyed endocrinologists in this study supported the continuous use of dexamethasone during pregnancy, a practice that contradicts current international guidelines which recommend discontinuation due to potential teratogenic effects and lack of proven benefit (16). Similar attitude gaps have been reported internationally; for example, a multi-country audit revealed variability in clinicians’ understanding of prenatal glucocorticoid use, with some European practitioners continuing off-guideline therapy based on outdated protocols (7). These findings suggest that clinical misconceptions about CAH management during pregnancy are not unique to China but reflect a broader issue in translating evolving evidence into practice. Addressing this requires not only general educational reinforcement, but also focused updates on guideline changes, consensus interpretation, and risk–benefit communication during prenatal counseling. The overall positive attitudes towards CAH underscore the potential for strengthening clinical practice through continuous medical education and awareness initiatives. By reinforcing knowledge on the less understood aspects of CAH and focusing on the dissemination of best practices, healthcare professionals can ensure even better patient care and outcomes (27). It is important to acknowledge that promoting awareness of CAH among primary care physicians, as believed by a majority of endocrinologists, can also contribute to more accurate and timely referrals, further enhancing patient care in CAH (28, 29). The relatively low confidence in managing fertility-related complications of CAH suggests a need for specialized training programs focused on reproductive health outcomes. This is particularly important given that poor hormonal control during reproductive years can lead to persistent anovulation, menstrual irregularities, testicular adrenal rest tumors, and irreversible infertility all of which substantially affect patients’ quality of life and limit their family planning options. Studies have shown that fertility rates in CAH patients are significantly lower than in the general population, but can be improved with early diagnosis, individualized steroid therapy, and multidisciplinary reproductive care (2, 3, 11). These findings underscore the need for proactive fertility management as a central component of long-term CAH care.

The results of this study offer insights into the clinical practices of Chinese endocrinologists concerning CAH. It is encouraging to note that a significant majority express the intention to prioritize the dosage of medication to avoid growth inhibition in CAH patients during their growth period, recommend regular screening for diagnosed patients, and provide disease education and psychological counseling during clinical diagnosis and treatment. Additionally, the commitment to update and adjust individualized treatment plans based on monitoring indicators and clinical presentation, as well as the recognition of the importance of multidisciplinary collaboration in CAH management, are in line with best practices for CAH care (30). However, a striking contrast is observed in the comparatively lower confidence in successfully identifying and diagnosing CAH. This discrepancy may suggest a gap in initial medical training or a lack of exposure to CAH cases, underlining the necessity for enhanced educational programs and clinical training in this area. To address this, medical curricula could be revised to include more in-depth coverage of rare endocrine disorders like CAH, and continuing medical education programs could be designed specifically to update practicing endocrinologists on the latest diagnostic techniques and treatment guidelines (29). Additionally, previous studies have highlighted the utility of algorithm-based diagnostic aids and integrated clinical decision support tools in improving diagnostic accuracy for rare endocrine disorders, including CAH. Integrating such tools into electronic medical record systems can assist frontline clinicians especially those in primary or secondary care with timely identification and appropriate referral (22). Given the diagnostic uncertainties observed in our study, such as the low proportion of endocrinologists confident in diagnosing CAH and frequent confusion with PCOS or other conditions, implementing standardized diagnostic algorithms may offer a practical and scalable solution for improving early recognition and differentiation.

The study’s correlation analysis highlights significant positive associations between knowledge and attitudes, knowledge and practice, and attitudes and practice. These findings underscore the interdependence of knowledge, attitudes, and practice in the context of CAH management among Chinese endocrinologists. To further improve clinical practice, it becomes imperative to recognize the importance of a holistic approach, where enhancements in knowledge and attitudes are likely to lead to more proactive and effective practices (31, 32).

The study has several limitations. Firstly, it relies on self-reported data from questionnaires, which may introduce response bias. Secondly, the study’s cross-sectional design only provides a snapshot of endocrinologists’ KAP at a specific point in time, making it challenging to establish causal relationships. Additionally, the study was conducted exclusively in China, which may limit the generalizability of the findings to other regions or populations. Lastly, the study utilized a convenient sampling method, distributing the survey via WeChat groups. Participation was voluntary, and the sample primarily consisted of endocrinologists who had prior contact with or specific interest in CAH. This approach may have introduced sampling bias, as the respondents tended to be mid-level or senior endocrinologists from tertiary hospitals. The study does not explore the potential impact of external factors, such as changes in guidelines or healthcare policies, on endocrinologists’ KAP regarding CAH. Fourthly, the questionnaire did not explicitly categorize questions into ‘classic’ and ‘non-classic’ CAH domains. Although several items addressed subtype-specific issues (e.g., differential diagnosis with PCOS for NCCAH, and testicular adrenal rest tumors for classic CAH), the overall KAP scores may not independently reflect the nuanced knowledge required for managing each distinct form. Future studies should employ more granular instruments to evaluate physician competency in managing classic versus non-classic variants separately.

The study underscores the importance of specialized training for endocrinologists, particularly early-career professionals, to enhance their knowledge and skills in diagnosing and treating congenital adrenal hyperplasia (CAH), with special emphasis on fertility preservation and reproductive health management, ultimately improving patient fertility outcomes and overall care. Further research should explore the specific impact of age and education on endocrinologists’ proficiency in CAH management, and longitudinal studies, qualitative research, and international comparisons can provide deeper insights for refining educational interventions and understanding regional variations.

While Chinese endocrinologists demonstrate generally positive attitudes toward CAH, significant knowledge and practice gaps persist, particularly in fertility management and diagnostic differentiation. The limited awareness of reproductive outcomes and low diagnostic confidence highlight an urgent need for targeted education to improve individualized fertility treatment options and reduce misdiagnosis in clinical practice.

## Data Availability

The original contributions presented in the study are included in the article/Supplementary material, further inquiries can be directed to the corresponding authors.
