# Supplementary material for: Knowledge, attitudes, and practices of Chinese endocrinologists on congenital adrenal hyperplasia: emphasis on fertility management and diagnostic challenges
Source: Front Public Health. 2026 Mar 27;14:1733159. doi: 10.3389/fpubh.2026.1733159 (PMC13066161; doi:10.3389/fpubh.2026.1733159)
Supplement: Supplementary file 1 [file Table_1.DOCX]

**Table S1. Distribution of scores by knowledge dimension**

|  | **a. Correct** | **b. Wrong** | **c. Unclear** |
| --- | --- | --- | --- |
| **1. Congenital adrenal hyperplasia (CAH) is an autosomal dominant genetic disease. (F)** | 230(48.4) | 212(44.6) | 33(6.9) |
| **2. CAH is caused by congenital defects in enzymes required for the synthesis of adrenal corticosteroids. (T)** | 448(94.3) | 3(0.6) | 24(5.1) |
| **3. 90%~95% of CAH cases are 17α-hydroxylase deficiency. (F)** | 211(44.4) | 231(48.6) | 33(6.9) |
| **4. CAH can lead to abnormally high levels of male hormones in females. (T)** | 429(90.3) | 22(4.6) | 24(5.1) |
| **5. In clinical practice, CAH needs to be ruled out before treating polycystic ovary syndrome (PCOS). (T)** | 448(94.3) | 5(1.1) | 22(4.6) |
| **6.** **CAH can cause masculinization of females, leading to gender misidentification. (T)** | 427(89.9) | 23(4.8) | 25(5.3) |
| **7.** **CAH can cause feminization of males, leading to gender misidentification. (T)** | 218(45.9) | 216(45.5) | 41(8.6) |
| **8. Most typical CAH cases result in adrenal malignancy. (F)** | 71(14.9) | 321(67.6) | 83(17.5) |
| **9.1 CAH may have the following symptoms and manifestations: — (1) vomiting, diarrhea, dehydration (T)** | 369(77.7) | 55(11.6) | 51(10.7) |
| **9.2 CAH may have the following symptoms and manifestations: — (2) asymptomatic or mild symptoms (T)** | 399(84.0) | 46(9.7) | 30(6.3) |
| **9.3 CAH may have the following symptoms and manifestations: — (3) sexual infantilism (T )** | 305(64.2) | 129(27.2) | 41(8.6) |
| **9.4 CAH may have the following symptoms and manifestations: — (4) precocious puberty (T )** | 360(75.8) | 82(17.3) | 33(6.9) |
| **9.5 CAH may have the following symptoms and manifestations: — (5) no pubic and axillary hair growth, delayed bone age (T)** | 270(56.8) | 171(36.0) | 34(7.2) |
| **9.6 CAH may have the following symptoms and manifestations: — (6) rapid height growth, advanced bone age (T)** | 334(70.3) | 101(21.3) | 40(8.4) |
| **9.7 CAH may have the following symptoms and manifestations: — (7) infrequent or absent menstruation (T)** | 442(93.1) | 15(3.2) | 18(3.8) |
| **9.8 CAH may have the following symptoms and manifestations: — (8) severe acne (T)** | 413(86.9) | 33(6.9) | 29(6.1) |
| **9.9 CAH may have the following symptoms and manifestations: — (9) underdeveloped testes (T)** | 355(74.7) | 84(17.7) | 36(7.6) |
| **9.10 CAH may have the following symptoms and manifestations: — (10) low blood potassium, fatigue, high blood pressure (T)** | 361(76.0) | 81(17.1) | 33(6.9) |
| **9.11 CAH may have the following symptoms and manifestations: — (11) low blood sodium, low blood volume, high blood potassium (T)** | 324(68.2) | 113(23.8) | 38(8.0) |
| **9.12 CAH may have the following symptoms and manifestations: — (12) elevated serum basal progesterone levels (T)** | 338(71.2) | 88(18.5) | 49(10.3) |
| **9.13 CAH may have the following symptoms and manifestations: — (13) dark skin and mucous membrane coloration (T)** | 387(81.5) | 51(10.7) | 37(7.8) |
| **10. Genetic testing is the gold standard for diagnosing CAH. (T)** | 434(91.4) | 27(5.7) | 14(2.9) |
| **11. In CAH laboratory tests, the best time for blood collection is usually around 8 a.m. (T)** | 421(88.6) | 20(4.2) | 34(7.2) |
| **12. In CAH laboratory tests, 17-α-hydroxyprogesterone measurement is an important clue for diagnosing CAH. (T)** | 437(92.0) | 15(3.2) | 23(4.8) |
| **13. In CAH laboratory tests, elevated adrenocorticotropic hormone (ACTH) and decreased cortisol (COR) are common manifestations. (T)** | 387(81.5) | 54(11.4) | 34(7.2) |
| **14. For CAH treatment, dexamethasone or hydrocortisone can be administered. (T)** | 425(89.5) | 18(3.8) | 32(6.7) |
| **15. The goals of modern adult CAH management are often aimed at preserving the patient's normal reproductive function to the maximum extent. (T)** | 361(76.0) | 87(18.3) | 27(5.7) |

**Table S2. Distribution of scores by attitude dimension**

|  | **a. Strongly agree** | **b. Agree** | **c. Neutral** | **d. Disagree** | **e. Strongly disagree** |
| --- | --- | --- | --- | --- | --- |
| **1. I believe that CAH is prone to misdiagnosis and underdiagnosis. (P)** | 308(64.8) | 141(29.7) | 24(5.1) | 1(0.2) | 1(0.2) |
| **2. I believe that 17-α-hydroxyprogesterone is an important clue for differentiating the diagnosis of CAH and PCOS. (P)** | 295(62.1) | 155(32.6) | 18(3.8) | 4(0.8) | 3(0.6) |
| **3. I believe that patients with confirmed CAH need lifelong oral corticosteroid treatment. (P)** | 223(46.9) | 155(32.6) | 74(15.6) | 21(4.4) | 2(0.4) |
| **4. I believe that female CAH patients can achieve natural pregnancy after individualized treatment. (P)** | 144(30.3) | 192(40.4) | 106(22.3) | 28(5.9) | 5(1.1) |
| **5. I believe that CAH patients need to use dexamethasone treatment throughout the pregnancy. (N)** | 93(19.6) | 156(32.8) | 135(28.4) | 68(14.3) | 23(4.8) |
| **6. I believe that choosing the right treatment timing, dosage, and regimen is particularly important for CAH treatment. (P)** | 338(71.2) | 119(25.1) | 14(2.9) | 2(0.4) | 2(0.4) |
| **7. I believe that although the clinical incidence of CAH is low, knowledge related to the diagnosis and treatment of CAH should still be emphasized. (P)** | 384(80.8) | 75(15.8) | 12(2.5) | 2(0.4) | 2(0.4) |
| **8. I believe that it is necessary to improve the awareness of CAH among primary care physicians. (P)** | 373(78.5) | 86(18.1) | 13(2.7) | 2(0.4) | 1(0.2) |

**Table S3. Distribution of scores by practice dimension**

|  | **a. Always will** | **b. Usually will** | | **c. Sometimes will** | **d. Rarely will** | | **e. Never will** |
| --- | --- | --- | --- | --- | --- | --- | --- |
| **1. For CAH patients in their growth period, I will emphasize the dosage of medication to avoid growth inhibition. (P)** | 289(60.8) | 159(33.5) | | 19(4.0) | 4(0.8) | | 4(0.8) |
| **2. I will recommend regular scrotal ultrasound screening/ovarian and adnexal imaging for diagnosed CAH patients. (P)** | 300(63.2) | 145(30.5) | | 22(4.6) | 4(0.8) | | 4(0.8) |
| **3. I would recommend surgical removal of testes to prevent tumor development for CAH patients whose social gender is female. (P )** | 211(44.4) | 156(32.8) | | 66(13.9) | 29(6.1) | | 13(2.7) |
| **4. I will provide disease education and psychological counseling to patients during clinical diagnosis and treatment. (P)** | 328(69.1) | 125(26.3) | | 16(3.4) | 4(0.8) | | 2(0.4) |
| **5. I will update and adjust individualized treatment plans based on monitoring indicators and clinical presentation. (P)** | 333(70.1) | 119(25.1) | | 19(4.0) | 2(0.4) | | 2(0.4) |
| **6. I will inform CAH patients that the treatment of CAH requires the collaboration of multidisciplinary physicians (such as endocrinologists, gynecologists, psychologists, geneticists, and obstetricians). (P)** | 360(75.8) | 98(20.6) | | 13(2.7) | 1(0.2) | | 3(0.6) |
|  | **a.Yes** | | **b.No** | | | **c.Unsure** | |
| **7. I can successfully identify and diagnose CAH in my clinical work. (OPEN)** | 256(53.9) | | 22(4.6) | | | 197(41.5) | |
